# An increase in plasma brain derived neurotrophic factor levels is related to n-3 polyunsaturated fatty acid efficacy in first episode schizophrenia: secondary outcome analysis of the OFFER randomized clinical trial

**DOI:** 10.1007/s00213-019-05258-4

**Published:** 2019-05-17

**Authors:** Tomasz Pawełczyk, Marta Grancow-Grabka, Elżbieta Trafalska, Janusz Szemraj, Natalia Żurner, Agnieszka Pawełczyk

**Affiliations:** 1 Department of Affective and Psychotic Disorders, Medical University of Lodz, ul. Czechoslowacka 8/10, 92-216 Lodz, Poland; 2 Child and Adolescent Psychiatry Unit, Central Teaching Hospital, Medical University of Lodz, ul. Pomorska 251, 92-213 Lodz, Poland; 3 Department of Nutrition Hygiene and Epidemiology, Medical University of Lodz, ul. Jaracza 63, 90-251 Lodz, Poland; 4 Department of Medical Biochemistry, Medical University of Lodz, ul. Mazowiecka 6/8, 92-215 Lodz, Poland

**Keywords:** Brain-derived neurotrophic factor, Neuroprotection, Fatty acid, Randomized clinical trial, Supplementation

## Abstract

**Rationale:**

N−3 polyunsaturated fatty acids (n−3 PUFA) influence multiple biochemical mechanisms postulated in the pathogenesis of schizophrenia that may influence BDNF synthesis.

**Objectives:**

A randomized placebo-controlled study was designed to compare the efficacy of a 26-week intervention composed of either 2.2 g/day of n−3 PUFA or olive oil placebo, with regard to symptom severity in first-episode schizophrenia patients. The secondary outcome measure of the study was to describe the association between n−3 PUFA clinical effect and changes in peripheral BDNF levels.

**Methods:**

Seventy-one patients aged 16–35 were enrolled in the study and randomly assigned to the following study arms: 36 to the EPA + DHA group and 35 to the placebo group. Plasma BDNF levels were assessed three times, at baseline and at weeks 8 and 26 of the intervention. BDNF levels were determined in plasma samples using Quantikine Human BDNF ELISA kit. Plasma BDNF level changes were further correlated with changes in the severity of symptoms in different clinical domains.

**Results:**

A significantly greater increase in plasma BDNF levels was observed in the intervention compared to the placebo group (Cohen’s *d* = 1.54). Changes of BDNF levels inversely correlated with change in depressive symptoms assessed using the Calgary Depression Rating Scale in Schizophrenia (Pearson’s *r* = − 0.195; *p* = 0.018).

**Conclusions:**

The efficacy of a six-month intervention with n−3 PUFA observed in first-episode schizophrenia may be related to an increase in BDNF levels, which may be triggered by the activation of intracellular signaling pathways including transcription factors such as cAMP-reactive element binding protein.

## Introduction

Neurodegenerative and neurodevelopmental hypotheses have been proposed to explain the pathogenesis of schizophrenia (SCZ). The latter proposes that SCZ develops due to disrupted developmental processes occurring in the brain, including the maturation and differentiation of neural cells, synaptic plasticity, and synaptogenesis. These pivotal neurodevelopmental processes are modulated by glial cells and glial-derived cytokines known as neurotrophins or growth factors (Nurjono et al. 2012). Brain-derived neurotrophic factor (BDNF) is one of the best studied and the most abundant members of the neurotrophin family in the adult brain. It plays a crucial role in processes known to be disrupted in SCZ, including neuronal migration, differentiation, and cell survival. Most previous studies have shown decreased peripheral levels of BDNF in schizophrenia patients, as reflected in the meta-analyses (Green et al. 2011; Fernandes et al. 2015). Postmortem studies have also revealed that the mRNA levels of BDNF, those of its receptor tyrosine kinase B (TrkB), and those of BDNF protein levels are decreased in the brains of patients with schizophrenia in specific regions like the prefrontal cortex and hippocampus, which are crucial for disease pathophysiology (Sugai et al. 2004; Thompson Ray 2011; Mohammadi et al. 2018b). Decreased serum and plasma levels of BDNF have also been documented in drug-naive and medicated schizophrenia patients, not only in cross-sectional and longitudinal studies but also in the meta-analyses (Green et al. 2011; Valiente-Gómez et al. 2014). Most studies have been carried out in chronic schizophrenia patients, but recent papers have described reduced peripheral BDNF levels in high clinical risk groups and patients with the first episode of schizophrenia (Martinez-Cengotitabengoa et al. 2016; Heitz et al. 2018). The authors propose that reduced levels of BDNF are indicative for the early stages of the development of psychotic disorders, with a noticeable drop in peripheral levels of BDNF seen just before psychosis development (Heitz et al. 2018). An association has also been observed between BDNF levels and severity of SCZ psychopathology (Nieto et al. 2013). Brain-derived neurotrophic factor has been shown to influence cognition and memory in healthy people but also in SCZ patients (Hori et al. 2014). Thus, BDNF has been proposed to function as a biomarker of cognitive deficit and a therapeutic parameter of cognitive improvement in SCZ patients (Galvez-Contreras et al. 2016; Hori et al. 2017). Reduced peripheral levels of other neurotrophins, including nerve growth factor (NGF) and vascular growth factor (VEGF), have also been described in SCZ patients. These observations have led to the formulation of the neurotrophin hypothesis of schizophrenia (SCZ), which postulates that the changes observed in the brains of SCZ patients occur as a result of disturbances in developmental processes involving neurotrophic factors (Lang et al. 2004).

There is continuing discussion about the role of polyunsaturated fatty acids (PUFAs) in the prevention of SCZ development (Amminger et al. 2010; McGorry et al. 2016). N−3 PUFA supplementation has been found to decrease psychopathology (Peet et al. 2002; Pawełczyk et al. 2016) and the cumulative doses of antipsychotics used in the first episode of the disease (Berger et al. 2008). Previous studies conducted in animal models of schizophrenia, as well as those performed in vitro, suggest that n−3 PUFA efficacy in SCZ may be related to the modulation of neurotrophic pathways (Rao et al. 2007; Fang et al. 2017). Although animal studies have yielded promising results suggesting n−3 PUFA efficacy in SCZ may be related to neuroplastic changes induced by BDNF, this relationship has not been studied systematically in a patient population so far. Moreover, previous studies have implied possible relationships between peripheral BDNF levels and the severity of schizophrenia symptoms (Li et al. 2016) or cognitive performance (Hori et al. 2014; Zhang et al. 2018) in patients diagnosed with schizophrenia. It was shown also that changes in peripheral BDNF level may be observed after PUFA supplementation, and these may be related to the severity of depressive symptoms in traumatized people (Matsuoka et al. 2015b). Hence, the aim of the present study was two-fold—(a) to assess and compare BDNF plasma level changes after 26-week supplementation with concentrated fish oil rich in n−3 PUFA or with a placebo (olive oil) and (b) to determine whether BDNF plasma level changes are related to improvements in psychopathology, disease severity, and patient functioning in those diagnosed with first episode of schizophrenia.

## Methods

The study presents the results of a secondary endpoint analysis of a randomized, double-blind n−3 PUFA intervention trial, the full details of which have been published elsewhere (trial registration: clinicaltrials.gov identifier NCT02210962). The detailed explanation of the study plan, description of the study sample, power calculation, inclusion and exclusion criteria, randomization process, study intervention, primary and secondary outcome measures and evaluation of chlorpromazine dose equivalents, medicines used during the study, and change in cumulative dose of antipsychotics used are given in detail elsewhere (Pawełczyk et al. 2015; Pawełczyk et al. 2016). The results of the primary outcome analysis, inter-rater reliability, adherence to medication, and adverse effects analysis have been described earlier (Pawełczyk et al. 2016) and are not replicated in the present paper.

### Study participants and procedures

All the participants were inpatients of the teaching and memorial hospitals located in the catchment area. The study group was composed of patients (1) aged 16–35 and (2) who were diagnosed with schizophrenia (first episode) according to the International Classification of Diseases, 10th version (ICD-10). The patients were excluded (1) if more than two years had passed since the first onset of positive symptoms; (2) if the patient had bleeding disorders; (3) had been using n-3 PUFA supplements within eight weeks or (4) was using anticoagulants for any reason; (5) was diagnosed with drug-induced psychosis, first-episode mania, organic disorders presenting with psychotic symptoms or intellectual disability; (6) if the patient had a history of head injury which required hospital admission, or any acute or unstable medical condition or one that could influence the results of the trial or affect their ability to take part in the trial; (7) if the patient was participating in another study.

The participants were part of the study group recruited for the OFFER randomized, placebo-controlled trial (Pawełczyk et al. 2016), the aim of which was to examine the efficacy of supplementation with concentrated fish oil containing 2.2 g of n−3 PUFA, i.e., eicosapentaenoic (1320 mg) and docosahexaenoic acid (880 mg) (EPA + DHA), in 71 drug-naive or early-treated first-episode schizophrenia patients. Seventy-one individuals were recruited for the study. Thirty six were randomly allocated to the EPA + DHA group, and 35 to the placebo group. The treatment groups did not differ with regard to demographic or baseline characteristics (Table 1). A detailed description and comparison of the potential confounding factors characterizing the study groups were described elsewhere (Pawełczyk et al. 2016).

**Table 1:** Baseline characteristics of participants^a^

Characteristic	EPA + DHA (*n* = 36)	Placebo (*n* = 35)	*p* value
Age, mean^b^ (SE)	22.93 (0.79)	23.06 (0.81)	0.894
Male sex, *n* (%)	19 (52.8)	23 (65.7)	0.268
Duration of untreated psychosis, mean^b^ (SE), mo	2.56 (0.69)	2.36 (0.59)	0.917
Family history of schizophrenia, *n* (%)	13 (36)	14 (40)	0.736
Years of education, mean^b^ (SE)	12.94 (0.45)	13.73 (0.52)	0.303
Marital status, *n* (%)			
Married	2 (6)	2 (6)	0.346
Single	34 (94)	31 (89)	
Divorced	0 (0)	2 (5)	
Place of living, *n* (%)			
Alone	5 (14)	4 (11)	0.573
With family	30 (83)	31 (89)	
Dormitory	1 (3)	0 (0)	
Employment, *n* (%)			
Employed	4 (11.1)	7 (20.0)	0.475
Not employed	17 (47.2)	18 (51.4)	
Sheltered workshops	1 (3)	0 (0)	
During education	14 (38.9)	10 (28.6)	
Tobacco use, *n* (%)	14 (39)	15 (43)	0.734
CDSS score, mean^b^ (SE)	8.08 (0.85)	6.78 (1.1)	0.263
CGI-S score, mean^b^ (SE)	5.91 (0.12)	5.71 (0.11)	0.297
GAF score, mean^b^ (SE)	26.12 (1.47)	27.6 (1.85)	0.461
PANSS score, mean^b^ (SE)			
Positive	24.79 (0.78)	22.87 (0.96)	0.081
Negative	25.91 (1.02)	25.6 (0.81)	0.791
General	49.17 (1.26)	48.11 (1.1)	0.712
Total	99.52 (2.15)	96.58 (1.84)	0.25
CPZ equivalent dose at baseline^c^, median (IQR) (mg)	0 (187.5)	0 (300)	0.256
CPZ equivalent dose at baseline^d^, mean (SE) (mg)	263.16 (21.46)	292.81 (33.07)	0.669

CDSS, Calgary Depression Scale for Schizophrenia; CGI-S, Clinical Global Impressions Severity Scale; PANSS, Positive and Negative Syndrome Scale; GAF, Global Assessment of Functioning Scale; *n*, number of participants in a group; CPZ, chlorpromazine; SE, standard error of the mean; IQR, interquartile range; mo, month; mg, milligram

^a^For a detailed description of the study groups, please refer to Pawełczyk et al. (2016)

^b^5% trimmed mean

^c^Entire population

^d^Participants on antipsychotics at baseline

A patient flow diagram presenting the history of participant inclusion, exclusion, and attrition at different stages of the study has been presented in detail previously (Pawełczyk et al. 2016).

The trial procedures were described verbally and in writing to all qualified patients. All participants gave written informed consent before study enrollment. Permission was obtained from parents or guardians for participants under 18 years of age. The study obtained authorization at the Ethics Committee of the Medical University of Lodz and was conducted under the Declaration of Helsinki.

### Study intervention

The active medicine was yellow gel capsules filled each with fish oil containing 0.33 g of EPA and 0.22 g of DHA. The daily dosage of four capsules provided 2.2 g of n−3 PUFA, i.e., 1.32 g/day of EPA plus 0.88 g/day of DHA. The placebo capsules contained olive oil, which is rich in monounsaturated fatty acids (73.9%) and contains only small amounts of polyunsaturated fatty acids (9.8%). Placebo capsules were prepared to resemble the active treatment in appearance and flavor. The placebo also contained a limited amount of fish oil to provide a comparable taste of the different capsules. Both placebo and active capsules contained an antioxidant, i.e., 0.2% alpha-tocopherol (vitamin E) to prevent the oxidation of fatty acids. Marinex International Sp z o.o. provided the study medication, both concentrated fish oil and placebo, obtained from Scandinavian Laboratories, Inc., Mt. Bethel, PA, USA. It was packed into numbered bottles and sent to the store of the Central Teaching Hospital of the Medical University of Lodz, Poland. Each bottle contained a fixed number of capsules of study medication or an equal amount of an olive oil placebo. Adherence to study intervention was monitored through patient/parent self-reporting and pill count at each medical appointment.

The use of benzodiazepines, Z-drugs, injectable forms of antipsychotics, antidepressants, mood stabilizers, and anticholinergic medications was allowed if clinically indicated, to increase the external validity of the study results and conform to schizophrenia therapy guidelines. Background antipsychotic and concomitant medication use were monitored throughout the study. The use of special diets or supplements, including other n−3 PUFAs, was not permitted throughout the study. Participants were assessed by a registered dietitian at the beginning of the study and encouraged to adhere to a well-balanced, continuous diet for the duration of the study.

### Outcome measures

Clinical scales were used to assess several domains of symptom severity and patient functioning at baseline and planned follow-up visits. After randomization, the participants underwent weekly assessments for four weeks and then at weeks 6, 8, 16, and 26. The primary outcome measure was the change in the total Positive and Negative Syndrome Scale (PANSS) (Kay et al. 1987) scores between baseline and 26 weeks. Secondary clinical outcome measures included the changes in the PANSS subscale scores (positive, negative, and general psychopathology), the Clinical Global Impressions (CGI) scale (Guy 1976), the Global Assessment of Functioning (GAF) (Jones et al. 1995), and the Calgary Depression Scale for Schizophrenia (CDSS) (Addington et al. 2014) scores between baseline and after 26 weeks of intervention. One of the secondary biochemical outcome measures was the change in PBMC telomerase level. Telomerase concentration was assessed three times—at baseline, 8 weeks, and 26 weeks after initiation of study intervention.

### Brain-derived neurotrophic factor level measurement

Blood samples were drawn in the morning between 7:00 and 10:00 a.m. following a fasting night. Fifteen milliliters of venous blood were drawn with subjects in the supine position after the subjects had been lying at rest overnight. Plasma was collected using EDTA as an anticoagulant. Specimens were centrifuged for 15 min at 1000 ×*g* at 2–8 °C within 30 min of collection. An additional centrifugation step of the separated plasma at 10,000 ×*g* for 10 min at 2–8 °C was used for complete platelet removal. Brain-derived neurotrophic factor (BDNF) levels were determined in plasma samples using Quantikine Human BDNF ELISA kit (R&D Systems Minneapolis, MN, USA) by measuring absorbance at 450 nm with correction at 540 nm. The assay was performed according to the manufacturer’s instructions.

### Statistical analyses

All analyses were conducted using an intent-to-treat (ITT) approach. Depending on the distribution of the dependent variables, the Student’s *t* test or Mann–Whitney *U* test was used to compare continuous variables of the treatment groups at baseline, while the Chi-squared test or Fisher’s exact test was used to examine differences in the categorical variables. Pearson’s correlation coefficient (Pearson’s *r*) was used to assess the linear association between change from baseline of clinical scores and change in BDNF level.

The missing data in the present study resulted from patient withdrawal or missed assessments. It therefore cannot be regarded as missing completely at random and, hence, must be modeled (Friedman et al. 2010). A conservative approach was taken to deal with missing values in our ITT sample. The chosen procedure assumed that BDNF concentration would have been maintained at the level that was observed during the previous visit the patient was assessed (last observation carried forward, LOCF).

The changes in BDNF level were assessed using a mixed model for repeated measures (MMRM) that included fixed-effect terms for intervention, visit, baseline score as a covariate, and an intervention-by-visit interaction term, using autoregressive heterogeneous covariance structure for within-patient correlation. Differences between treatment groups were reported using least-squares (LS) means with standard error (SE). Planned contrasts were conducted to assess differences between study groups at two time points—t1 (8 weeks) and t2 (26 weeks). Cohen’s *d* effect sizes were estimated as the difference in LS mean change scores between treatment and placebo divided by the model estimate of the pooled standard deviation. All statistical analyses used two-sided tests, with statistical significance set at alpha = 0.05.

## Results

### Study sample

Seventy-one individuals were recruited for the study; 36 were randomly assigned to the EPA + DHA group and 35 to the placebo group. The treatment groups were similar regarding demographic variables and baseline characteristics (Table 1). One of the 36 (1.8%) participants from the EPA + DHA group terminated the intervention early and withdrew his consent. Three patients of the 36 (8.3%) from the EPA + DHA group were lost to follow-up and did not attend follow-up evaluations. Two patients of the 35 (5.7%) allocated to the placebo group were lost to follow-up; one moved out of the region and the other did not attend any follow-up assessments. Therefore, the 26-week follow-up intervention was completed by 65 participants; 32 (88.9%) from the EPA + DHA group and 33 (94.3%) randomized to the placebo group. The difference in drop-out rate between groups was not statistically significant (Fisher’s exact test; *p* = 0.674).

At the time of enrollment, 43 participants (60.6%) were antipsychotic naive and 17 had fewer than nine days of medication. Among those medicated, the mean duration of antipsychotic therapy was 14 days (SE = 3.3). All but five patients were treated with antipsychotics for less than six weeks before enrollment. Study groups were not significantly different according to the frequency of antipsychotic-naive patients enrolled (Chi-squared test; Chi^2^ = 1.139; *p* = 0.286). The groups were not different regarding the duration of antipsychotic therapy before trial inclusion (Mann–Whitney *U* test; *Z* = 1.201; *p* = 0.230) nor concerning baseline chlorpromazine equivalent dose (Table 1). All patients were treated with antipsychotics after 26-week intervention. Daily consumption of energy and PUFA was determined at baseline using the Polish version of the Food Frequency Questionnaire (Dehghan et al. 2012). Dietary consumption of energy and PUFA was not significantly different between the groups at baseline (Table 1).

### BDNF levels

**Fig. 1:**
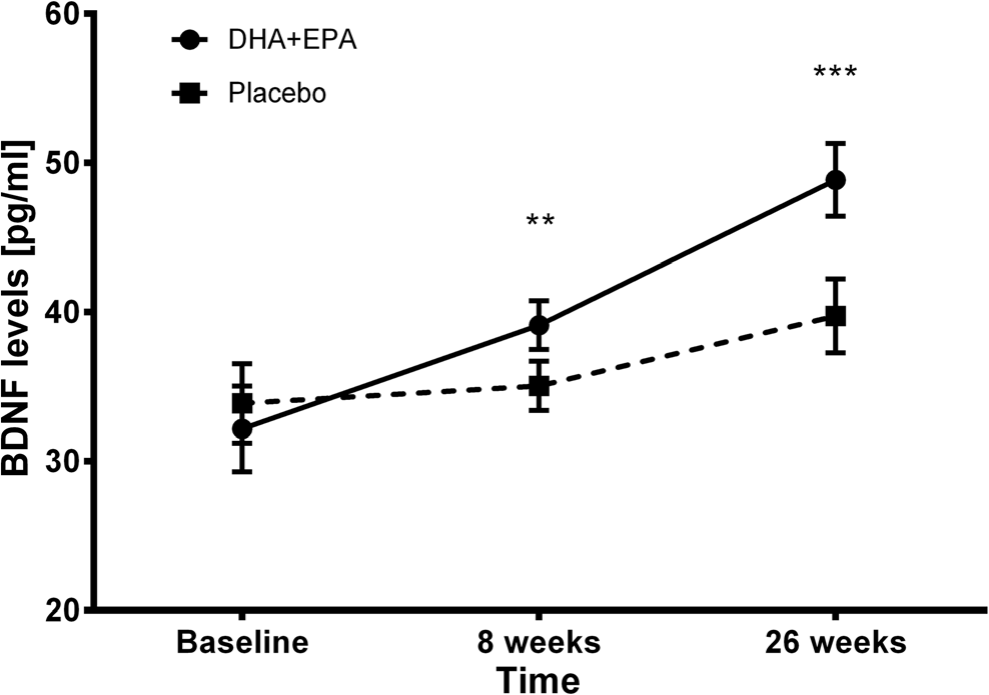
Plasma BDNF levels at baseline, week 8, and week 26 in the study arms. Points and error bars represent the means and 95% confidence intervals. Differences between groups: **p < 0.01; ***p < 0.001. Estimated marginal means at time points (8 and 26 weeks) were adjusted for BDNF level at baseline (BDNF0), which was entered into a model as a covariate and is evaluated at the following value: BDNF = 33.01

The Mixed Model for Repeated Measures analysis (MMRM) was used to assess differences between groups regarding the BDNF levels. Significant increases in BDNF concentrations were observed in both groups during the study (paired-sample Student’s *t* test, EPA + DHA: *t* = 4.737, df = 35, *p* < 0.001; placebo: *t* = 4.768, df = 34, *p* < 0.001) and were significantly higher in the EPA + DHA group than in the placebo BDNF (Fig. 1). The analysis of contrasts from MMRM revealed significant differences between groups regarding BDNF plasma levels. Significant intervention-by-visit interaction was observed (*t*_(119,244)_ = − 2.449; *p* = 0.016; 95% CI − 9.119 to − 0.966). The mean change of BDNF plasma level from baseline was significantly higher in the EPA + DHA than in the placebo group. The observed effect (*d* = 1.54) can be considered as high according to the classification of effects provided by Cohen (1988). The least-squares mean changes and mean differences between groups in change scores at week 26 compared to baseline are presented in Table 2. An analysis of contrasts between the study groups in different time points revealed significant differences in BDNF levels both at eight weeks (*t* = 3.552, df = 69, *p* = 0.001) and at 26 weeks (*t* = 5.317, df = 69, *p* < 0.001). Mean BDNF levels with 95% confidence intervals are presented in Fig. 1.

**Table 2:** Differences in BDNF level change from baseline to week 26 in the study groups

Variable	Change from baseline to week 26^a^, mean (SE)		Least-squares mean difference^b^ (95% CI)	Effect size^c^
	EPA + DHA (*n* = 36)	Placebo (*n* = 35)		
BDNF level (pg/ml)	10.986	4.388	6.598^*^	1.54
	(0.724)	(0.735)	(4.55–8.646)	

SE, standard error; CI, confidence interval

^*^p < 0.001

^a^Estimated marginal means adjusted for BDNF level at baseline (BDNF t_0_), which was entered into the model as a covariate and is evaluated at the following value: BDNF t_0_ = 33.01

^b^Based on the contrast from mixed-models repeated-measures analysis

^c^Difference in change from baseline in units of standard deviations of change

### Correlations with clinical outcome measures

A significant negative correlation was seen between the change from baseline to week 26 for plasma BDNF levels and changes from baseline to week 26 regarding the score of the Calgary Depression Scale for Schizophrenia (CDSS). Correlation coefficients for significant associations are displayed in Table 3. According to Cohen (1988), the magnitude of the observed significant correlation between BDNF and CDSS can be considered as small to medium. The remaining associations between the changes of BDNF levels and changes of the other clinical assessments were found to be insignificant (Table 3).

**Table 3:** Correlations between changes from baseline to 26 weeks in clinical measures and change from baseline in plasma BDNF level in the study population (*N* = 71)

Variable change from baseline to 26 weeks	Pearson’s *r*	*p* value^a, b^
PANSS score		
Total	− 0.099	0.225
Positive	0.033	0.69
Negative	− 0.067	0.417
General	− 0.109	0.185
CDSS	− 0.195	0.018*
GAF	0.088	0.285
CGI-S	− 0.074	0.399

CDSS, Calgary Depression Scale for Schizophrenia; CGI-S, Clinical Global Impressions Severity Scale; PANSS, Positive and Negative Syndrome Scale; GAF, Global Assessment of Functioning Scale; *N*, number of observations in a population

Significant correlations were marked with an asterisk; *p < 0.05

^a^Two-sided asymptotic test probability for Pearson’s *r* correlation coefficient

^b^Due to exploratory nature of the analyses, no adjustments for multiple comparisons were conducted (Savitz and Olshan 1995)

## Discussion

### Comparison with previous studies

This is the first RCT study that describes changes in peripheral BDNF levels in schizophrenia patients supplemented with PUFA. Therefore, the results obtained in the present study cannot be compared with previous data. Only one human study has assessed the changes in peripheral BDNF levels during PUFA supplementation. Matsuoka et al. (2015a) carried out such a randomized double-blind controlled study with patients vulnerable to post-traumatic stress disorder (PTSD) and depression after injury who underwent intervention composed of mainly DHA plus small amounts of EPA or placebo for 12 weeks after injury. The authors observed that the increase in serum levels of mature BDNF and precursor pro-BDNF correlated negatively with the severity of depression, but not PTSD symptoms. The authors concluded that increased BDNF and pro-BDNF may exert a protective effect by minimizing depression severity but were unable to prevent PTSD development (Matsuoka et al. 2015a). The authors did not analyze the probable mechanism of n−3 PUFA effects on BDNF levels.

Human studies regarding n−3 PUFA relationships with BDNF levels are scarce. However, many animal studies have observed that dietary supplementation with n−3 PUFA resulted in changes in BDNF concentration and content. Fang et al. (2017) report that supplementation with n−3 PUFA could prevent neuronal loss and cognitive impairment via enhancement of the cAMP-response element-binding protein (CREB)/BDNF/tyrosine kinase B (TrkB) pathway in an animal model of schizophrenia. Activation of CREB/BDNF/TrkB pathway related to long-term n−3 PUFA supplementation has also been observed in neutral aging rats which also correlated with cognitive improvement (Gao et al. 2016). Another group of researchers has found that dietary EPA regulates BDNF function, attenuates glial activation, and normalizes hippocampal n−3 and n−6 PUFA profiles in a rodent model of neuroinflammation (Dong et al. 2017). Adult rats fed energy-matched diets enriched in saturated fatty acids for two weeks showed impaired place recognition memory compared with rats consuming a diet rich in n−3 PUFA (Beilharz et al. 2016). However, the two groups in the study did not differ according to hippocampal BDNF gene expression. Elsewhere, both supplementation with n−3 PUFA and direct hypothalamic injection were found to potentially induce hypothalamic cell neurogenesis which correlated with BDNF expression (Nascimento et al. 2016).

In addition, reduced neuronal degeneration has been observed in transgenic mice endogenously synthesizing n−3 PUFA, and this has been attributed to the high n−3 PUFA levels present, acting via BDNF signaling (Bak et al. 2015), and n−3 PUFA has also been found to bestow a preventive effect associated with prefrontal cortex BDNF reduction in an animal model of schizophrenia induced by ketamine administration (Zugno et al. 2015). Moreover, supplementation with fish oil rich in n−3 PUFA through three generations of rats was found to result in increased hippocampal BDNF mRNA expression and lower reactive oxygen species generation (Trevizol et al. 2015b; Trevizol et al. 2015a).

The consumption of a trans-fat diet has been found to have the opposite effect on BDNF expression and reactive oxygen species (ROS) generation. N−3 PUFA supplementation can reduce the effects of amphetamine regarding the hippocampal level of BDNF mRNA and ROS in rats subjected to the animal model of mania. Another experiment carried out in frogs has shown that long-term consumption of a diet deficient in n−3 PUFA results in structural neural abnormalities—fewer dendrite branches, shorter dendritic arbor, and lower postsynaptic cluster number and density. Moreover, changes in neuronal morphology correlated with a decrease in the levels of BDNF mRNA and mature protein in the brain. Interestingly, switching to n−3 PUFA was found to restore the structural changes observed in animals fed with a PUFA-deficient diet (Igarashi et al. 2015). In contrast, however, Keleshian et al. (2014) do not report any such amelioration of the deleterious effects observed following NMDA administration in PUFA-supplemented animals with an adequate n−3 PUFA content. However, dietary PUFA deficiency resulted in the reduction of BDNF levels. Transcriptomic analysis of brains of murines fed on fish oil-enriched diets revealed suppression of BDNF along with a reduction in oxidative stress and amyloid beta production and an increase in somatostatin activation (Hammamieh et al. 2014).

Gama et al. report an increase in serum BDNF levels in rats subjected to a ketamine model of schizophrenia after n−3 PUFA supplementation, together with a reduction of positive, negative, and cognitive symptoms in n−3 PUFA-fed animals, which they speculate might have been related to increases in serum BDNF level (Gama et al. 2012). However, in contrast to other studies (Trevizol et al. 2015a; Zugno et al. 2015), n−3 PUFA supplementation was not found to have any influence on BDNF mRNA expression in the prefrontal cortex, hippocampus, or striatum of the animals.

Rao et al. have observed that 15-week n−3 PUFA dietary deprivation in rats decreases frontal cortex DHA content and reduces BDNF expression, CREB transcription factor activity, and p38 mitogen-activated protein kinase (MAPK) activity. The authors suggest that these mechanisms may contribute to the therapeutic efficacy of PUFA in brain neurodegenerative diseases, such as schizophrenia (Rao et al. 2007).

Peripheral and central BDNF reductions have been found to be related to cognitive dysfunction (Hori et al. 2014) and positive and negative symptoms (Xiu et al. 2009). However, these results are not consistent (Atake et al. 2018). Serum BDNF levels have been related to hippocampal volumes in first-episode schizophrenia patients (Rizos et al. 2014). Earlier findings attributed the response to antipsychotics to the presence of the BDNF Val66Met polymorphism (Nikolac Perkovic et al. 2014). However, this relationship was questioned in a recent meta-analysis (Cargnin et al. 2016). Nevertheless, although no n−3 PUFA supplementation studies assessing neurotrophin profile have been conducted in schizophrenia patients, the animal studies described strongly support the results of the present study, which indicate increases in BDNF peripheral levels after n−3 PUFA supplementation in first-episode schizophrenia patients.

Interestingly, in the present study, a significant increase in BDNF levels was also observed in the placebo group treated with antipsychotics. Previous studies have reported non-conclusive results regarding the influence of antipsychotics on peripheral BDNF levels (Huang 2013). It has been revealed that serum BDNF levels are higher in patients treated with clozapine than those treated with risperidone or typical antipsychotics (Tan et al. 2005; Grillo et al. 2007; Xiu et al. 2009; Rizos et al. 2010), suggesting serum levels of BDNF can vary depending on the type of drug used. Nevertheless, Rizos et al. have stated that serum BDNF levels were significantly increased in the patients treated with olanzapine compared to those treated with haloperidol, risperidone, or amisulpride (Rizos et al. 2010). Similarly, higher BDNF levels have also been observed in SCZ patients treated with clozapine than those treated with typical antipsychotic agents, with the peripheral level of BDNF correlating strongly with clozapine dose (Grillo et al. 2007). Pedrini et al. (2011) have also reported serum BDNF levels to be significantly correlated with clozapine daily dose, but not the typical antipsychotic daily dose; however, other longitudinal studies have not found any increase in serum BDNF levels in patients with schizophrenia after antipsychotic treatment (Pirildar et al. 2004; Rizos et al. 2010; Yoshimura et al. 2010; Kudlek Mikulic et al. 2017). In contrast, increased levels of serum BDNF have been observed after electroconvulsive and antipsychotic therapy in schizophrenia patients (Li et al. 2016). However, the most recent meta-analysis found peripheral BDNF levels to be consistently increased in plasma, but not serum, following antipsychotic treatment, irrespective of the patient’s response to medication (Fernandes et al. 2015). Decreased peripheral BDNF levels have been more consistently observed in first-episode drug-naive patients, which has been interpreted as a potential pathogenic mechanism of disease development. To summarize, the increase in BDNF levels observed in the placebo group in the present study does not resolve the controversy over the relationship between antipsychotic therapy and peripheral BDNF levels. It is possible that the increase in BDNF levels observed in the placebo group reflects the early stage of the disease, where the processes related to the BDNF pathway are more active and susceptible to change than in chronic schizophrenia patients.

A significant correlation was observed in the present study between changes in BDNF plasma levels and changes in the severity of depressive symptoms. Depressive symptoms have been reported to be associated with peripheral BDNF level in humans following n−3 PUFA supplementation (Matsuoka et al. 2015b) and in animal models (Venna et al. 2009; Pudell et al. 2014; Choi and Park 2017). However, studies in populations of patients diagnosed with major depression have not revealed any association between peripheral BDNF levels and the severity of depressive symptoms (Jevtović et al. 2011; Bouckaert et al. 2016; Caldieraro et al. 2017). However, the studies could lack the power to detect a signal.

The most recent meta-analysis (Zhou et al. 2017) has revealed significant increases of peripheral BDNF levels following antidepressant treatment in patients diagnosed with major depression. Significant differences between antidepressant medications were detected, showing sertraline to be the most effective agent in increasing peripheral BDNF levels. It was also found that time modifies the effect of BDNF release, with higher increases of peripheral BDNF levels being observed after longer antidepressant therapy. Another interesting finding was that antidepressant therapy appeared to have significant effects on BDNF level in serum but not in plasma, although this could be due to the high heterogeneity of the results; serum appeared to be more reliable than plasma for measurement of BDNF levels. In addition, a temporal correlation was also found between antidepressant therapy, serum BDNF levels, and the effect of treatment. The negative correlation between the change in peripheral BDNF levels and the change in depressive symptoms observed in the present study implies that BDNF may be a mediator of the antidepressant effect induced by n−3 PUFA in patients with first-episode schizophrenia.

### Mechanism of action

A potential molecular mechanism of n−3 PUFA modulation of BDNF levels may be related to CREB pathway activation, which has been shown to be influenced by n−3 PUFA in the animal studies described. CREB is a key transcription factor and one that regulates plenty of intracellular signaling pathways, including neuroprotection, neuroinflammation, cellular growth, and survival (Bak et al. 2015). CREB transcriptional properties require phosphorylation, which may result from activation of multiple kinase systems, such as phosphokinase A (PKA), phosphokinase C (PKC), mitogen-activated protein kinase (MAPK), and Ca^2+^ calmodulin-dependent kinase IV (CaMK IV). These enzymes are able to phosphorylate the Ser-133 residue of the CREB transcription factor, thereby increasing its binding to the active sites in the nucleus and promoting the transcription of dependent genes including BDNF.

Dietary deprivation of n−3 PUFA for 15 weeks in rodents resulted in increased depression and aggression scores; this has been related to decreased phosphorylation of p38 MAPK, which in turn has been suggested to lead to decreased activation of CREB and reduced BDNF expression (Rao et al. 2007). In a study in which rats were subjected to traumatic brain injury (TBI), the increase in oxidative stress and learning impairment was marked by a decrease in BDNF, and supplementation of DHA counteracted the effects of TBI and normalized BDNF as well as CREB levels (Wu et al. 2004). A study with primary astrocytes demonstrated that DHA was able to induce BDNF expression through a pathway involving p38 MAPK (Rao et al. 2007). Ser133 phosphorylation of CREB has been found to be increased in transgenic mice (fat-1) that are able to synthesize n−3 PUFA endogenously (Bak et al. 2015). Collectively, these findings support the idea that n−3 PUFA enrichment can stimulate BDNF expression in animal models via activation of CREB by increasing the Ser133 phosphorylation induced by p38 MAPK. MAPK and CREB signaling abnormalities have been implicated in schizophrenia, since they play a crucial role in a plethora of pathophysiologic processes that are disrupted in schizophrenia, such as neuroinflammation, apoptosis, cell survival, cell growth, and oxidative stress (Crisafulli et al. 2015; Igolkina et al. 2018; Mohammadi et al. 2018a).

Abnormal activation of the CREB and MAPK signaling pathways has been observed in the frontal cortex of post-mortem brains obtained from patients with an established diagnosis of schizophrenia (Funk et al. 2012). Hence, abnormalities in the MAPK and CREB pathways have been suggested to be involved in the pathology of schizophrenia. Moreover, fluoxetine has been also found to regulate MAPK p38 activation, which induces BDNF synthesis and is related to an antidepressant effect (Tiraboschi et al. 2004). The serum protein levels of the BDNF pathway have been implicated in depression and antidepressant treatment efficacy (Jiang et al. 2017). To summarize, n−3 PUFA has been shown to modulate BDNF expression via CREB and p38 MAPK phosphorylation, which is related to mood and cognitive improvement. This mechanism may account for the changes in plasma BDNF levels and their correlation with the reduction in depressive symptomatology observed in the present study.

### Limitations

The present study holds some limitations that need to be acknowledged before conclusions can be formulated, the main one being a lack of any objective measure of adherence since it was not possible to assess the concentration of n−3 PUFA in the red blood cells of study participants. However, because this study measured peripheral rather than central nervous system levels of BDNF, it is unknown how the observed results translate exactly to brain changes or pathology. Antibody affinity methods used to measure BDNF levels were not specific enough to discriminate between the proBDNF molecule and mature BDNF. However, there may be differences between their molecular mechanisms of action.

The strengths of the study include its randomized, placebo-controlled design, blinding and its inter-rater reliability testing. Another strength and novel aspect is the composition of n−3 PUFA used, i.e., a 3:2 mixture of EPA and DHA, which has not yet been used in patients with first-episode schizophrenia; this dosage of PUFA supplementation was higher than that used in earlier studies but low enough to ensure the safety of intervention.

## Conclusion

Our findings indicate that 26-week supplementation with n−3 PUFA added to antipsychotics in first-episode schizophrenia patients was related to a significant increase in peripheral BDNF levels; this was also observed in the second arm of the study, in which patients received placebo (olive oil) and antipsychotics. Changes of BDNF levels within the whole population were inversely related to the severity of depressive symptoms. Taking into consideration the several and complex roles of BDNF, the results of the present study encourage exploration of new fields of research in patients with schizophrenia, such as the relationship between p38MAPK/CREB/BDNF/TrkB pathway activation and (a) disease activity and symptom severity, (b) intensity of the neuropathological processes disrupted in schizophrenia, such as cell growth, survival and apoptosis, neuronal plasticity, oxidative stress and neuroinflammation, and (c) the processes of neurodegeneration and neurogenesis, especially in hippocampi, and the possible role of n−3 PUFA in modulating these processes. Moreover, this study offers the possibility for sharing some of the mechanisms of action by both antipsychotics and n−3 PUFA, including modulation of BDNF levels. Thus, p38MAPK/CREB/BDNF/TrkB may constitute a novel pharmacological target in schizophrenia that can be explored in further studies.
